# Integrating technology in mental healthcare practice: A repeated cross-sectional survey study on professionals’ adoption of Digital Mental Health before and during COVID-19

**DOI:** 10.3389/fpsyt.2022.1040023

**Published:** 2023-02-16

**Authors:** Milou Feijt, Yvonne de Kort, Joyce Westerink, Joyce Bierbooms, Inge Bongers, Wijnand IJsselsteijn

**Affiliations:** ^1^Human-Technology Interaction Group, Eindhoven University of Technology, Eindhoven, Netherlands; ^2^Philips Research, Eindhoven, Netherlands; ^3^TRANZO Digital, Tilburg University, Tilburg, Netherlands; ^4^Erasmus School of Health Policy and Management, Erasmus University Rotterdam, Rotterdam, Netherlands; ^5^Mental Healthcare Eindhoven, Eindhoven, Netherlands

**Keywords:** COVID-19, Digital Mental Health, eMental Health, mental healthcare professionals, blended care, videoconferencing, mental healthcare, adoption of innovations

## Abstract

As a consequence of the outbreak of the COVID-19 global pandemic in the spring of 2020, large-scale social distancing measures were implemented, resulting in the forced adoption of online or digital forms of psychological treatment. This sudden transition to digital care offered a unique opportunity to investigate if and how this experience impacted mental healthcare professionals’ perceptions and use of Digital Mental Health tools. The current paper presents findings of a repeated cross-sectional study consisting of three iterations of a national online survey in the Netherlands. This survey contained open and closed questions on professionals’ adoption readiness, frequency of use, perceived competency, and perceived value of Digital Mental Health collected in 2019 (before the pandemic), in 2020 (after the first wave), and in 2021 (after the second wave). The inclusion of data gathered prior to the COVID-19 pandemic offers a unique window to assess how professionals’ adoption has developed through this transition from voluntary to mandatory use of Digital Mental Health tools. Our study also re-assesses the drivers, barriers, and needs of mental healthcare professionals after having gained experience with Digital Mental Health. In total, 1,039 practitioners completed the surveys (Survey 1: *n* = 432, Survey 2: *n* = 363, and Survey 3: *n* = 244). Results indicate that compared to the period before the pandemic, there was a particularly large increase in use, competency, and perceived value regarding videoconferencing. Small differences were also found for some other basic tools that were crucial to ensure the continuation of care, such as e-mail, text messaging, and online screening, but not for more innovative technologies, such as virtual reality and biofeedback. Many practitioners reported to have gained skills regarding Digital Mental Health and experienced several benefits of it. They expressed the intention to continue with a blended approach, using Digital Mental Health tools in combination with face-to-face care, focused on situations in which they found it to have specific added value, such as when clients are unable to travel. Others were less satisfied with the technology-mediated interactions and remained more reluctant to future use of DMH. Implications for broader implementation of Digital Mental Health and future research are discussed.

## 1. Introduction

In the past decades, the integration of technology in mental healthcare has been extensively researched. Digital Mental Health (DMH), also referred to as e(Mental) Health can be defined as “the use of information and communication technolog*y* (ICT)—in particular, the many technologies related to the Internet—to support and improve mental health conditions and mental healthcare” (1). DMH has been associated with several unique benefits, such as increased accessibility of mental healthcare, reduced travel time and costs, flexibility, and enhanced autonomy of clients (2, 3). Multiple systematic reviews have compared the efficacy of DMH to traditional face-to-face interventions – either in stand-alone or in blended forms – and generally found evidence for equivalent therapeutic outcomes (4, 5). Other studies have focused on whether online treatment allows for sufficient levels of therapeutic alliance or rapport, generally considered a key component of successful therapeutic outcomes, finding satisfactory results (6). However, at the same time, various studies point to the contrast of these encouraging findings with the persistently low uptake of technologies in actual daily clinical practice (7–9).

Much previous research has been dedicated to clarifying the difficulties of implementing eMental Health and found that, amongst other factors, the adoption by practitioners (10, 11) is crucial, as they are generally less receptive of DMH than clients (12, 13). In these studies, adoption has been shown to be a multifaceted concept. A well-known conceptualization has been offered by Rogers (14), who describes adoption as a staged process that comprises necessary knowledge and skills, acceptance, implementation in daily procedures, actual use, and evaluation. A related term is adoption readiness, i.e., “*the extent to which a professional is ready to use DMH (i.e., has a positive attitude, is motivated, and possesses the necessary skills and knowledge)”* (15). Adoption readiness specifically captures the individual characteristics of professionals’ adoption and so is less dependent on organizational, legal, and other factors external to the practitioner.

Studies on the adoption of DMH by professionals indicate various barriers toward online therapy. Important barriers include lack of therapists’ competence and training, technological problems, and concerns about impoverished communication and the detrimental effects this has on the quality of the therapeutic relationship (2, 3, 16). Other main barriers are characteristics of clients that do not fit with DMH, not knowing how to deal with crisis situations, privacy and security issues, and loss of therapeutic control (2, 3, 16). Interestingly, in contrast with the concerns about negative effects on the therapeutic interaction, multiple studies also describe that practitioners report enhancement and intensification of the therapeutic relationship as a prominent advantage of DMH, because it allows for more frequent moments of contact in between sessions (3). Another important factor influencing practitioners’ acceptance is their belief that DMH tools can be effective and useful (2). Furthermore, lowering the barrier to care, flexibility, efficiency, and convenience due to reduced travel time are found to be the most prominent benefits that could result in a positive attitude toward DMH (16).

For long, the adoption of DMH in practice remained at a stable and low level (7, 9). The advent of the COVID-19 pandemic, however, necessitated a drastic change for mental healthcare practice. Physical distancing measures forced the majority of mental health practitioners to almost instantly transfer their practice to remote modalities, leading to the large-scale use of online mental healthcare services (17, 18). While acknowledging the great burden the pandemic and its social and physical consequences caused for both practitioners and clients, this period also offers an opportunity to investigate the lived experience of DMH tool use by a much broader group of users – both therapists and clients – than have been exposed to such tools to date.

The experience gained by using DMH tools during the pandemic might have a positive effect on practitioners’ adoption readiness for two main reasons first, earlier work showed that providing knowledge about DMH can increase practitioners’ intention to use DMH (19). In addition, although studies directly comparing attitudes before and after use of DMH are relatively scarce, those that did generally showed an increase in adoption, specifically higher acceptance and ease of use, and fewer concerns about low efficacy and negative consequences for the therapeutic relationship (2). At the same time, however, the gained experience might also expose on which aspects DMH falls short compared to face-to-face treatment. In addition, one must also note that, during COVID-19, the use of DMH was mainly imposed on practitioners by organizational or governmental regulations. Research has shown that the impulse created by initial usage of an innovation is not necessarily a predictor of future use intentions (20), hence, use of DMH tools may fall back to previous low levels as soon as practitioners are free to decide themselves again. Furthermore, due to the sudden shift to online means, the technological infrastructure was often not yet in place. This resulted in practical issues such as a lack of the necessary hard- and software and problems with the stability of the internet connection, on the side of the practitioner as well as the client (18).

In the past 2 years, a multitude of studies has been conducted to investigate professionals’ experiences with DMH during COVID-19. A concise synthesis of these studies indicates that practitioners in general were positive about their use of DMH, but also that this appreciation was mostly in light of the crisis situation, as DMH offered the possibility to at least continue their practice in some way (21). Besides the particular benefit of continuation of care, other reported advantages mostly seem to overlap with those described in pre-pandemic studies. The experienced difficulties concerning the treatment process were also similar to previous studies; participants predominantly reported to miss non-verbal cues through online communication and struggled with how to deal with crisis situations remotely (18, 22). In addition, especially at the beginning of the transition, there were major issues with access to the required technological devices and stable internet connections (18, 21, 22). Also, practitioners reported higher levels of fatigue due to communicating through videoconference tools for multiple consecutive hours (18). Furthermore, procedures for registration of treatment hours and guidelines to adhere to privacy standards were often not in place (21, 22). Generally, this improved during the course of the pandemic (21).

Most of the work focusing on the effects of COVID-19 on DMH adoption has been conducted in an *ad hoc* fashion, swiftly responding to the unexpected and unprecedented situation (21). Therefore, these studies generally assess practitioners’ perceptions in that moment, and rely on retrospective accounts of professionals to compare their current (i.e., during the pandemic) use, acceptance, and skills to the situation pre-COVID. People, however, are found to be biased when they are asked to recall their past; for example, they tend to report past attitudes and feelings that are more in line with their current perceptions (23, 24). Therefore, one cannot draw strong conclusions on the extent to which reported changes in adoption truly have occurred. In addition, many of the studies presented either quantitative or qualitative data. While quantitative studies have the advantage of enabling the recruitment of large, representative samples and performing statistical comparisons, it is difficult to determine *why* these outcomes are found. Qualitative data does have the benefit of providing in-depth insights but then cannot be easily generalized across a population or be used to test for differences between samples. Combining both types of data allows for triangulation of the results, to inspect if they corroborate each other and to discover potential contradictions, and to use results of one method to facilitate interpretation of the other, resulting in a more in-depth understanding of the entire body of data (25).

### 1.1. Current study

The current study presents a repeated cross-sectional survey study, analyzing data of three iterations of an online questionnaire with open and closed questions, assessing the adoption of DMH by mental healthcare professionals. With this study, we aim to answer the following research questions:

1. How have general DMH adoption readiness and use, competency, and perceived value of various DMH tools developed from before to well into the COVID-19 pandemic?
2. What are the drivers, barriers, and needs of mental healthcare professionals after having gained forced experience with DMH tools, and which are the most important factors motivating or impeding future use?

To address these questions, the current research will compare data collected before the pandemic to that collected over the course of the COVID-19 pandemic with the aim to explore how this experience has affected professionals’ adoption of DMH. To our knowledge, no study to date has directly compared self-reported data gathered before the pandemic to data obtained after two waves of COVID-19. In addition, whereas previous work has mainly focused on either DMH in general or one specific DMH tool – predominantly videoconferencing – the current study probes different DMH tools separately, allowing to differentiate professionals’ adoption for tools of varying levels of innovativeness.

Regarding the quantitative measurements of DMH use, our main hypotheses are as follows: we expect that frequency of use of DMH tools has increased between Survey 1 (2019) and both Survey 2 (2020) and Survey 3 (2021) due to the COVID-19 pandemic and its associated social distancing measures (e.g., lockdown) that occurred in between. Resulting from this gained experience, we also expect an increase in competency and perceived value between Survey 1 and both Survey 2 and Survey 3. Last, we hypothesize that these increases will lead to an increase in general adoption readiness between these time points.

In addition, the combination of quantitative and qualitative data allows us not only to test whether any changes have occurred, but also provides insight into why differences have arisen. We combine this with data on the most important experienced drivers and benefits to further understand what motivates or impedes future use of DMH. Together, this provides new information on what actually drives change in practitioners’ adoption of DMH. In turn, this could guide directions toward sustainable implementation of DMH in practice and eventually extend therapists’ treatment possibilities.

## 2. Materials and methods

### 2.1. Design

**FIGURE 1:**
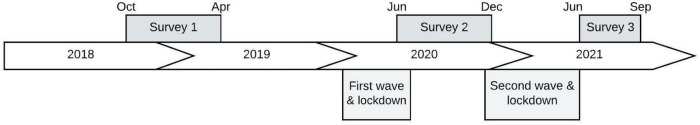
Timeline of surveys and waves of the pandemic.

The current study involved a repeated cross-sectional research study, consisting of three iterations of an online survey – disseminated nationally in the Netherlands – with closed and open questions on mental healthcare professionals’ adoption of DMH. The entire survey can be found in Supplementary Table 1. Data were collected at three time points: Survey 1 was held between October 2018 and April 2019 (before the pandemic), Survey 2 between June and December 2020 (after the first wave of the pandemic), and Survey 3 between June and September 2021 (after the second wave of the pandemic). During these latter two periods, the lockdowns were (temporarily) ended and most professionals were allowed to meet their clients in person again. In most organizations, there were strict rules regarding physical hygiene, maintaining 1.5-m distance, wearing face masks when moving around, and staying home when one had symptoms that were related to COVID-19 or had been in contact with someone that was infected by COVID-19. Because of these last two regulations, sometimes sessions still were conducted remotely. Figure 1 presents a timeline indicating the measurement periods of the surveys and the waves of the pandemic.

### 2.2. Participants

The target sample comprised of mental health care professionals with a range of experience in using DMH and sampled across a variety of mental healthcare professions and mental health care domains. For each iteration, the same recruitment procedure was followed: we contacted several large mental health care organizations in the Netherlands. In total, six mental health care institutions recruited participants either through email or intranet. Furthermore, an announcement was placed in the newsletters and websites of three national professional associations of psychologists: the Dutch Institute for Psychology, the Dutch Association of Health Psychologists, and the Dutch Association of Independent Psychotherapists. Last, several independent practitioners were contacted directly through the authors’ networks. Upon completion of the survey, participants could sign up for a raffle in which 12 gift vouchers of 50 Euros were allotted per survey as a reward for their participation.

In total, 1,039 participants completed the surveys, with slightly decreasing numbers for each consecutive survey (Survey 1: *n* = 432, Survey 2: *n* = 363, Survey 3: *n* = 244). Most of the participants (63%) were working in secondary mental healthcare. Other prevalent mental healthcare domains concerned forensic institutions (10%), basic mental healthcare (8%), and children and youth (7%). There were no significant differences in demographic characteristics between the three surveys.^1^ Table 1 presents further details on the demographic data of the three samples.

**TABLE 1:** Demographic data for the first (*n* = 432), second (*n* = 363), and third (*n* = 244) survey, including gender, age, and profession.

Characteristic	Survey 1	Survey 2	Survey 3
**Gender**			
Male	144 (33%)	95 (26%)	62 (25%)
Female	288 (67%)	268 (74%)	181 (74%)
**Age, in years**			
Mean (range)	41 (20–69)	39 (18–70)	42 (21–69)
**Profession**			
Clinical/counseling psychologists and psychotherapists	125 (29%)	133 (37%)	92 (38%)
Psychiatric nurses	140 (32%)	111 (31%)	70 (29%)
Social work	102 (24%)	68 (19%)	49 (20%)
Expressive therapists (e.g., creative arts therapist and psychomotor therapist)	13 (3%)	8 (2%)	5 (2%)
Physicians (e.g., psychiatrist, general practitioner, and neurologist)	27 (6%)	22 (6%)	15 (6%)
Other (e.g., researcher and team manager)	25 (6%)	21 (6%)	13 (5%)
**Years of clinical experience**			
Mean (range)	16 (0–43)	14 (0–47)	16 (0–45)
**Previous DMH education**			
Yes	159 (37%)	142 (39%)	90 (37%)
No	273 (63%)	221 (61%)	154 (63%)

### 2.3. Measures

#### 2.3.1. Frequency of use, competency, and perceived value

Frequency of use, competency, and perceived value were measured through self-developed items. They were probed for each of 12 DMH tools: e-mail, text-messaging/chat, educational website, online modules, social media, videoconferencing, monitoring apps, client portal, online screening, wearables and biofeedback, virtual/augmented reality, and domotics. *Frequency of use of DMH* was measured for each of these 12 DMH tools on 5-point Likert scales ranging from 1 (“almost never”) to 5 (“almost every day”). *Perceived competency* was measured regarding the same 12 DMH tools on 5-point Likert scales ranging from 1 (“not at all competent”) to 5 (“very competent”). Last, *Perceived value* of DMH was probed for the same 12 DMH tools on 5-point Likert scales from 1 (“not valuable”) to 5 (“very valuable”). For each of these measures, we also calculated a mean score over all 12 tools.

#### 2.3.2. Adoption readiness

Adoption readiness was measured through the eMental Health Adoption Readiness (eMHAR) Scale (15). The eMHAR Scale consists of 15 statements about the practitioner’s readiness to adopt DMH. It distinguishes three underlying factors: benefits and perceived applicability of EMH, EMH personal innovativeness, and EMH self-efficacy, but for the current analyses we only used the full-scale score. The items are rated on 5-point Likert scales ranging from 1 (“strongly disagree”) to 5 (“strongly agree”). Scores are obtained by first reverse scoring the negatively phrased items and then calculating the unweighted mean of all items. Higher scores reflect a higher level of adoption readiness. Cronbach’s alpha was 0.89 for this study.

#### 2.3.3. Barriers, drivers, and needs

In Surveys 2 and 3, we also added questions on barriers, drivers, and needs experienced by professionals while using DMH. These items were based on a previous review (3) and two qualitative studies conducted during the first months of the COVID-19 pandemic (18, 26). In total, practitioners were presented with 14 barriers, 9 drivers, and 10 needs, and for each category also an open item “other” in which they could report experiences that were not listed. For each item, practitioners could indicate whether they had experienced it (“yes”) or had not experienced it (“no”).^2^

#### 2.3.4. Practitioners’ perceptions and experiences on their use of DMH

Because of the outbreak of COVID-19 between Surveys 1 and 2, we added several open-ended questions to Survey 2 and 3 on reasons for increases or decreases in use and perceived value of DMH. Furthermore, in both Survey 2 and 3, we asked which tools, elements, or aspects of DMH they would like to continue using after the pandemic. Additionally, Survey 3 included a question about their expected use after all restrictions would be lifted and their reasons for having this expectation. This set of open-ended questions provided the qualitative data for this study.

#### 2.3.5. Background questions

Finally, the survey included basic demographic questions and several items regarding the characteristics of participants’ everyday clinical practice, such as years of professional experience, which psychological symptoms were most prominent amongst their clients, which psychological interventions they provided, and whether and which kind of training they had received regarding DMH.

The time required to complete the survey was approximately 15 min for Survey 1 and 20 min for Surveys 2 and 3 due to the added questions.

### 2.4. Data analytic strategy

#### 2.4.1. Changes in adoption variables

To analyze changes in use, perceived value, competency, and adoption readiness between surveys, we applied Welch’s *F*-tests with Games-Howell *post-hoc* tests. Welch’s *F*-tests have been shown to perform better with unequal sample sizes and with unequal variances, which are common in psychological research, while losing little power when the assumption of homogeneity of variances is met (27). Games-Howell *post-hoc* tests use Welch’s *t*-statistics to compare differences between each pair of means with appropriate adjustment for multiple testing. To estimate the relative sizes of the differences, effect sizes for each pairwise comparison were calculated with Cohen’s *d*_*s*_, which includes Bessel’s correction for bias in the estimation of the population variance (28, 29).

#### 2.4.2. Barriers, drivers, and needs

To facilitate the analysis of the experienced barriers, drivers, and needs reported on in Surveys 2 and 3, we first applied exploratory factor analysis to these items with the aim to condense them into a smaller number of factors and facilitate interpretation and increase power. We included all three categories, 32 items in total, into one analysis to allow underlying relationships between these factors to surface. Because the barrier “Type of treatment/client group” in Survey 2 was considered ambiguous in hindsight, this item was reformulated in Survey 3 and therefore left out of this joint analysis.

For the exploratory factor analysis, we used a robust weighted least squares estimator for categorical variables with an Oblimin oblique rotation. We chose for an oblique rotation method because many constructs in social sciences can be expected to correlate with each other (30–32). Moreover, orthogonal and oblique factors will produce similar results in the (unlikely) case that the factors are actually uncorrelated (33). The root mean square error of approximation (RMSEA) and the comparative fit index (CFI) were used to assess model fit; RMSEA < 0.07 and CFI > 0.96 indicate an acceptable model fit (30). These fit indices have shown to be more reliable in determining the number of factors to extract than parallel analyses when analyzing dichotomous variables (34). Loadings >0.30 were perceived as indicating practical significance (30). In case of cross-loadings (i.e., factor loadings exceeding 0.30 on multiple factors), the item was assigned to the factor that was most appropriate based on theoretical interpretation. Last, an unweighted mean score of each factor was calculated for each participant to be used in further analyses.

#### 2.4.3. Practitioners’ perceptions and experiences

The qualitative data on perceptions and experiences of practitioners were analyzed using a reflexive thematic analysis approach (35, 36). This approach consists of six phases: first, familiarization with the data by reading through it, followed by the second phase of generating codes with an inductive orientation, that is, with a bottom-up approach where the researcher starts the analytic process from the data without basing this on previous ideas and theories. Third, these codes were analyzed to generate themes. Fourth, these themes were defined and fifth revised, after which in the sixth phase the final themes and their subthemes were reported. The qualitative analysis was performed by the first author. The extracted codes and themes were reviewed by a second co-author, and commonalities and differences were discussed until agreement was reached.

Participants often presented their own explanations of how various aspects of adoption were causally related, which provided indications of how these themes and their subthemes could be related to each other. We used these to structure our descriptive model of themes and subthemes and included suggested connections as tentative interrelations.

#### 2.4.4. Analytic tools

The exploratory factor analysis was performed using MPlus 8.6 (37). SPSS 25 was used for all other statistical analyses. The qualitative data analysis was conducted through MAXQDA VERSION 22.1.

## 3. Results

### 3.1. Adoption readiness, use, perceived value, and competency

For all tools together, significant differences emerged between the three surveys for frequency of use (*F*_2,626_._143_ = 33.579, *p* < 0.001), competency (*F*_2,636_._17_ = 10.15, *p* < 0.001), and perceived value (*F*_2,623_._18_ = 6.403, *p* = 0.002), but not for adoption readiness (*F*_2,603_._82_ = 1.958, *p* = 0.142). *Post hoc* comparisons showed that use and competency were significantly higher in Survey 2 (*p* < 0.001 and *p* = 0.005) and 3 (both *p*’s < 0.001) compared to Survey 1, but not between Survey 2 and 3. The perceived value of DMH tools was higher in Survey 3 compared to Surveys 1 (*p* = 0.003) and 2 (*p* = 0.011), but not between Survey 1 and 2. All descriptive statistics can be found in Supplementary Table 2.

**FIGURE 2:**
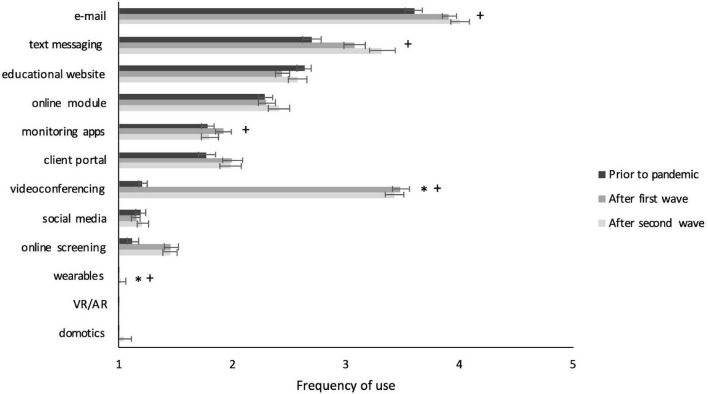
Frequency of use of individual DMH tools per survey. Significant effects with Cohen’s *d* > 0.25 are indicated with * for comparison between Survey 1 and 2 and with + for comparisons between Survey 1 and 3. Error bars present the standard error of the mean.

**FIGURE 3:**
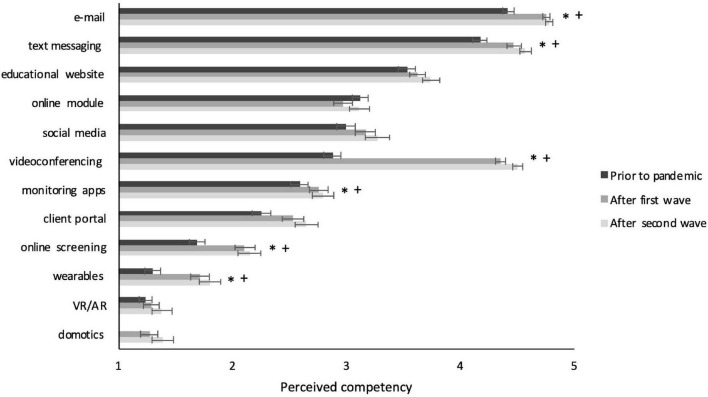
Perceived competency with individual DMH tools per survey. Significant effects with Cohen’s *d* > 0.25 are indicated with * for comparison between Survey 1 and 2 and with + for comparisons between Survey 1 and 3. Error bars present the standard error of the mean.

**FIGURE 4:**
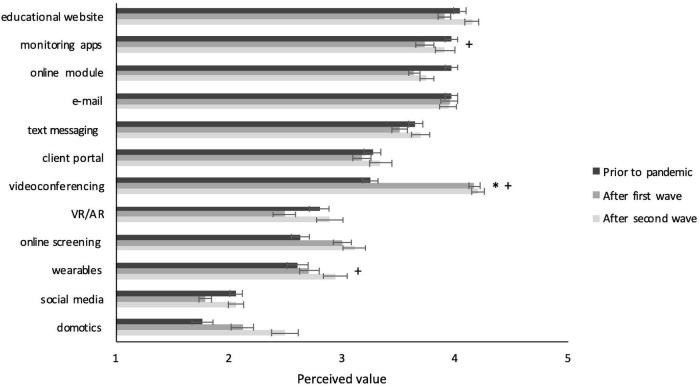
Perceived value of individual DMH tools per survey. Significant effects with Cohen’s *d* > 0.25 are indicated with * for comparison between Survey 1 and 2 and with + for comparisons between Survey 1 and 3. Error bars present the standard error of the mean.

Next, we inspected whether significant differences also existed between surveys in use, competency, or perceived value per specific tool (see Figures 2–4). This analysis clearly showed large differences for videoconferencing, in frequency of use (*F* = 562.565, *p* < 0.001), competency (*F* = 188.681, *p* < 0.001), and perceived value (*F* = 78.92, *p* < 0.001). *Post hoc* comparisons showed that on all these measures scores for videoconferencing were higher for Surveys 2 and 3 compared to Survey 1 (all *p*’s < 0.05 and Cohen’s *d* between 0.8 and 2.2), but not between Survey 2 and 3. Furthermore, use and competency significantly increased – be it to a much smaller extent – for basic treatment tools such as e-mail (*F* = 7.68, *p* = 0.001 and *F* = 21.187, *p* < 0.001), text messaging (*F* = 10.935, *p* < 0.001 and *F* = 12.23, *p* < 0.001), and online screening (*F* = 11.413, *p* < 0.001 and *F* = 9.652, *p* < 0.001), whereas perceived value only increased for online screening (*F* = 8.383, *p* < 0.001). Again, *post hoc* comparisons showed that all these values increased from Survey 1 to both Survey 2 and 3 (all *p*’s < 0.05 and Cohen’s *d* between 0.25 and 0.5). Differences on other basic tools such as client portal and monitoring apps were not significant or practically negligible (i.e., Cohen’s *d* < 0.25). Differences on innovative tools such as wearables and VR/AR were also non-significant or small, except for domotics, for which use (*F* = 596.355, *p* = 0.001), competency (*F* = 10.664, *p* < 0.001), and perceived value (*F* = 12.087, *p* < 0.001) did increase significantly, with medium effect sizes for the differences between Surveys 1 and 3 (all *p*’s < 0.05 and Cohen’s *d* between 0.3 and 0.4). The full statistics of these analyses can be found in Supplementary Table 3.

### 3.2. Barriers, drivers, and needs

Exploratory factor analysis of the 33 individual items on experienced barriers, drivers, and needs indicated a nine-factor solution to be optimal in terms of fit indices, theoretical interpretation, and extent to which it approached a simple structure. In this process, one item [i.e., “Not able to perform physical exercises (e.g., use of whiteboard, roleplaying)”] was removed due to theoretical incoherence with the factors. Another item (i.e., “Technological helpdesk that is readily available”) was moved to another factor despite a loading of 0.218 based on theoretical fit and because the alphas of concerning factors remained the same or even improved with this modification. The final model showed very good fit indices (χ^2^_222_ = 272.4, *p* < 0.001; CFI = 0.98, RMSEA = 0.019). The table with factor loadings of the final model can be found in Supplementary Table 4.

Four of the nine factors concerned a combination of barriers and needs that were related: a set of barriers and needs related to a stable internet connection were combined into the factor *Connection quality*. The factor *Equipment availability* pertained to barriers and needs regarding the availability of the required devices and software. Barriers and needs regarding the workload and how to register online hours were covered by the factor *Daily work process*, and barriers and needs regarding adherence to the privacy standards by the factor *Privacy*. Two factors consisted of only experienced barriers: *Empathic interaction* referred to issues with the ability to establish an empathic interaction online and missing non-verbal cues and *Client-related barriers* contained barriers regarding lack of skills, equipment, or an appropriate home environment of clients, or resistance to receive treatment through DMH. The factor *Implementation needs* concerned needs regarding the further implementation of DMH such as the extension of available software, training in DMH, and exchange of best practices with peers. Last, two factors covered the drivers: *Practical benefits* concerned drivers such as reduced travel time, increased flexibility, more efficient sessions or team meetings and *Client-oriented benefits* included advantages such as clients becoming more active with DMH and more open in their own environment.

**FIGURE 5:**
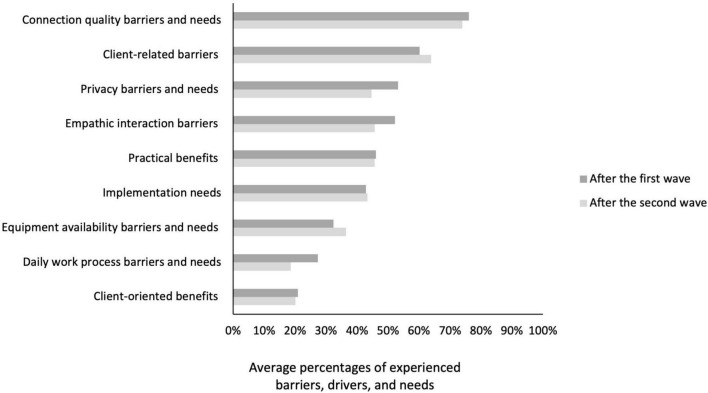
Average percentages of experienced barriers, drivers, and needs by practitioners during Survey 2 and 3.

Among the factors that covered barriers and/or needs, *Connection quality barriers and needs* were experienced most often (76% in Survey 2 and 74% in Survey 3), followed by *Client-related barriers* (60% and 64%), *Empathic interaction barriers* (52% and 46%), and *Privacy barriers and needs* (53% and 45%). Less frequent were *Implementation needs* (both 43%), *Equipment availability barriers and needs* (32% and 36%), and *Daily work process barriers and needs* (27% and 19%). Regarding drivers, *Practical benefits* were experienced more (both 46%) than *Client-oriented benefits* (21% and 20%). These results are also presented in Figure 5.

### 3.3. Qualitative results: Practitioners’ experiences and perceptions

**FIGURE 6:**
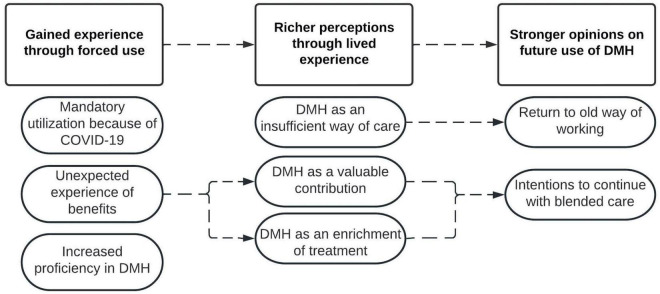
Model of derived (sub)themes of practitioners’ perceptions and experiences regarding DMH. Squares present themes, ovals present subthemes, and dotted lines present proposed relationships between (sub)themes.

Three main themes and seven subthemes were derived from the responses to the open-ended questions, the main themes being: gained experience through forced use, richer perceptions through lived experience, and stronger opinions on future DMH use. A descriptive model presenting these (sub)themes and their proposed relations is shown in Figure 6.

#### 3.3.1. Gained experience through forced use

##### 3.3.1.1. Mandatory utilization because of COVID-19

Even though some participants stated they already used DMH in their daily practice well before the pandemic, approximately half of the participants indicated that their use of DMH was imposed by their organization, because of regulations related to COVID-19 mandated by the government to work from home and minimize face-to-face contact as much as possible.

“More use of eHealth because of regulations requiring us to work from home as much as possible.” (s3p26)

##### 3.3.1.2. Unexpected experience of benefits

Almost half of the participants indicated to find DMH tools more valuable than before the pandemic. Many reported that even though their use of DMH was out of necessity, they were introduced to several benefits of DMH, often against their expectations. Because they experienced these advantages first-hand now, they became aware and more convinced of the added value of DMH.

“Only now, it has become apparent which advantages using eHealth brings.” (s2p300)

Several of them also expressed that DMH worked much better than they had anticipated and that many elements of treatment can be conducted online just as well as in traditional face-to-face settings. Some also reported that online sessions allowed for higher quality of contact with clients than expected and that they could be just as effective as offline sessions.

“Beforehand I was skeptical about its efficacy, afraid that it would impede the therapeutic relationship or that the treatment would be disrupted. Neither is the case.” (s2p162)

##### 3.3.1.3. Increased proficiency in DMH

Additionally, half of the participants expressed that their knowledge of and skills with DMH increased as a result of their experience during the COVID-19 pandemic. Several mentioned that they had become more aware of the possibilities of DMH and now knew how to apply it in their treatments. Some elaborated that previously they either did not have or take the time to become proficient in DMH, but now they simply had to because it was the only way to still be able to provide care to their clients.

“By doing, I have become much more familiar with DMH and obtained skills.” (s3p210)

“I became much more proficient in videoconferencing, which provides the flexibility to apply this later (that is, after COVID-19), if necessary.” (s2p107)

#### 3.3.2. Richer perceptions through lived experiences

##### 3.3.2.1. DMH as a valuable contribution

The benefit of DMH that was mentioned most often was that it allowed for continuation of care. This was particularly mentioned in the context of COVID-19: DMH enabled the only possible form of treatment in times when there was a lockdown in place and face-to-face sessions were not allowed or when people were in quarantine.

“In times in which all treatments for clients came to a standstill, I was very happy that some therapies/sessions could continue *via* eHealth.” (s2p105)

Even though this benefit was mainly reported in relation to the pandemic, many practitioners expressed that they viewed this as an important benefit of DMH for post-pandemic times as well, in situations when clients would be unable to travel, for example, due to illness or other obligations.

Similar to what we found in the quantitative data, the second most mentioned benefit of DMH concerned time savings and specifically reduced travel time, mainly for the client and in some cases for practitioners as well. Related to this, many practitioners appreciated the increased flexibility of planning appointments, especially when it concerned short moments of contact, evaluations, or staff meetings with multiple people or from external parties.

“The lack of travel time for clients as well as myself offers enormous flexibility in your treatment, and I feel this is a significant benefit.” (s3p62)

Another frequently reported driver was the lower threshold for contact, which had beneficial effects in various ways. First, for some clients, communicating through online means lowered the barrier to reach out, making treatment more accessible.

“For some clients it is easier to take the step towards treatment.” (s2p65)

Other practitioners explained that because online contact was easier to establish, it allowed for more frequent short moments of contact, which extended their options for communication and in turn improved the therapeutic relationship.

“You can have more short moments of contact with you clients, which actually facilitate recovery.” (s3p195)

In the same line, sometimes practitioners’ use of DMH was motivated by requests of clients because they preferred online contact based on practical (e.g., reduced travel time) or psychological (e.g., feeling more comfortable in their own home environment) reasons.

##### 3.3.2.2. DMH as an enrichment of treatment

In line with the relatively low frequency of *Client-oriented benefits* in the quantitative data, drivers related to changes in clients’ behavior were less frequently reported. Still, approximately five percent of the participants expressed they experienced that clients became more engaged in their own treatment, and that DMH stimulated clients to be more autonomous and proactive. They viewed this as a unique asset of DMH.

“Clients become more empowered by this, they are activated to take ownership.” (s2p116)

Related, a similar number of practitioners mentioned that they felt DMH broadened their treatment possibilities, providing new intervention elements they could offer to their client and allowing to tailor their care to the specific needs of individual clients. These participants considered DMH as an enrichment of their treatment.

“I find that it is a huge enrichment of my options as a therapist, such as the employment of online modules, the messaging feature, and the possibility of having short moments of contact through videoconferencing.” (s3p62)

##### 3.3.2.3. DMH as an insufficient way of providing care

Despite these positive remarks, there were also a substantial number of participants who mentioned they were not satisfied with online therapy and felt it had no added value for their client group, or worse, that it harmed their clients’ wellbeing and recovery process.

Participants regularly reported that their client group was not suitable for DMH, for example, because they did not possess the necessary devices or skills, or that their clients themselves strongly preferred to have contact in person rather than *via* videoconferencing.

“Both before and during the COVID-19 pandemic, my clients did not possess the devices nor the skills to use eHealth. Because they are not able to do that, I continued with face-to-face contacts, just as before the COVID-19 period.” (s2p353)

Notably, there was considerable variation in the type of client groups that practitioners mentioned to be difficult to treat *via* online means. Generally, practitioners working in in-patient care reported that DMH had less added value since all client contact was in-person anyway, although some mentioned that they valued online modules because they allowed clients to work on their recovery independently and that they used videoconferencing to communicate with a client’s social network. Regarding other client groups, opinions were rather mixed: elderly, young children, clients on the autism spectrum, suicidal clients, and group sessions were mentioned several times as not suitable for DMH, although for each of these groups there were also positive experiences from practitioners expressing that online forms worked well.

Some practitioners voiced strong opinions that face-to-face contact was essential to truly connect on a deeper level. Those who elaborated on this suggested that this was partly caused by missing non-verbal cues, such as small facial details or body movements when communicating online.

“Deeper connections are made face-to-face, and not *via* a screen.” (s2p66)

#### 3.3.3. Stronger opinions on future use of DMH

In their accounts, it became clear that, through their use, practitioners had formed a much more concrete view on whether, when and how they would apply DMH in a post-pandemic future than before, based on their own experiences.

##### 3.3.3.1. Intentions to continue with blended care

Approximately three quarters of the practitioners explicitly mentioned that they intended to continue using DMH tools to some extent, mostly videoconferencing. This group concerned the practitioners who generally perceived DMH as a valuable supplement or enrichment of treatment. They explained that they felt this way because they experienced particular situations in which DMH could be valuable.

“I think that being introduced to it because of COVID-19 has made us, mental healthcare professionals, but also our clients, more aware of when it can be of added value.” (s3p40)

Most often, practitioners reported to still have a preference to provide treatment primarily in person and then add online elements to this, that is, provide a blended form of treatment, with the aim to accommodate the specific preferences, abilities and needs of their clients. In other words, for these practitioners, DMH has now become an option that could be integrated in their care practice. In particular, videoconferencing was mentioned often as a tool that practitioners intended to continue using, either to meet with clients or for staff meetings.

##### 3.3.3.2. Return to old way of working

For a second, smaller group of participants, however, DMH experiences during the lockdown strengthened their dislike of DMH. Almost a fifth of the practitioners explicitly stated that they would return to their old way of working, involving solely face-to-face sessions, as soon as possible. These participants generally viewed DMH as an insufficient way of providing care, and so they were only prepared to use DMH during the pandemic because there was no alternative.

“I started using eHealth, mostly videoconferencing, because I had to. As soon as face-to-face contact was possible, I quickly went back to doing this again.” (s3p235)

In this group, barriers related to client characteristics and difficulties with the therapeutic interaction were particularly prominent. Interestingly, the quantitative data showed that these barriers were the most frequently experienced barriers in the entire sample, but from the qualitative data this appeared to be a crucial factor determining to abandon future use for only part of the participants. Notably, in contrast to the quantitative findings, technological barriers such as connection issues or lacking necessary devices were rarely mentioned as the premier reasons to refrain from using DMH, nor were issues with privacy or performance or productivity pressures.

## 4. Discussion

### 4.1. Principal findings

The current study presents findings of a repeated cross-sectional study involving three iterations of an online survey on mental healthcare professionals’ adoption of DMH over a period before and during COVID-19. As expected, we found a sharp increase in the use of DMH after the onset of the pandemic, but only for relatively basic DMH tools, particularly videoconferencing, that were crucial to enable continuation of the treatment process, and not for more innovative technologies also subsumed under the label of DMH (e.g., Virtual Reality and biofeedback). Furthermore, the necessity of providing care remotely urged practitioners to obtain the skills required to use these essential tools, reflected in increased perceived competency in this specific set of technologies.

Increases in perceived value were only present for the most essential tools during the pandemic: videoconferencing and online screening. Despite these changes in use, competency, and perceived value, we did not find a change in general adoption readiness scores at the group level. However, professionals’ qualitative responses did reflect altered perceptions, for many in positive but for others in negative directions, and practitioners experienced multiple advantages and disadvantages of DMH. Most reported benefits pertained to the continuation of care during COVID-19 or the increased flexibility, but some practitioners also mentioned positive effects for the quality of their care, mainly because it lowered the threshold for contact and broadened their options for treatment. Most frequent experienced barriers included technological issues, however barriers concerning client characteristics and problems to establish a satisfactory therapeutic interaction seem more important factors that determine future use. Although some practitioners were strengthened in their dislike of DMH, almost three quarters of the participants expressed the intention to continue with a blended approach; combining face-to-face sessions with DMH tools in specific situations in which they consider it to have specific added value.

The majority of our quantitative and qualitative findings validate and extend previous work. Our results confirm the large-scale uptake of remote care that was found for other countries as well (17), though only for the tools that were necessary to enable continuation of care, which was particularly salient for videoconferencing tools, but also for e-mail, text messaging and online screening. As expected, we found that not only did the use of these necessary tools increase, so did practitioners’ competency with them. Qualitative accounts also described how practitioners gained skills through experience. We can conclude that increased use of specific DMH tools, even though born out of necessity, has indeed led to increased competency in these tools. Obviously, since these are correlational data, we cannot assume a unidirectional, causal relationship; having the skills to work with a particular tool will likely increase chances of using it again, and vice versa.

A clear association between use and perceived value, in contrast, was not found, except for videoconferencing and to a lesser extent for online screening, which both showed substantial increases in use during the pandemic compared to before the pandemic. These results indicate that having skills and experience – although perhaps necessary – are not a sufficient condition for an increase in perceived value, especially when usage is not voluntary. As supported by the qualitative responses, several practitioners felt certain online tools fell short compared to providing the intervention in a face-to-face manner, regardless of their proficiency in using that tool, and as a consequence did not perceive it as valuable.

Contrary to our expectations, and despite the increases in use, competency, and to some extent perceived value of specific tools, we did not find a change in general adoption readiness scores. This demonstrates that the underlying mechanism of change in adoption readiness is more complex than merely competency in and perceived value of specific tools. Indeed, adoption readiness also includes several other individual factors, such as motivation, proactivity, personal innovativeness, and the perceived applicability of DMH in mental healthcare in general (15). Our findings indicate that these factors were not significantly affected by the mandatory use. This lack of change in adoption readiness might also explain why professionals did not become receptive to a much wider range of technologies, including more innovative tools: only the tools that were necessary to enable continuation of care were applied, but their usage did not trigger the use of more advanced DMH tools (i.e., virtual reality, wearables). Based on this finding, one could say that practitioners have gained skills on a “need to know” basis: they obtained the skills that were required to enable continuation of the treatment process when face-to-face interventions were not a viable option anymore. Considering the extremely challenging circumstances it is not surprising that such a situation does not lend itself to experimentation with other, potentially more novel tools. Lack of time and resources to acquire the necessary skills has often been reported as a main barrier to the adoption of DMH already before the pandemic (19), and one can assume that a global health crisis and its significant ramifications within mental healthcare practice will only have exacerbated such issues. However, it could also be that an increase of receptiveness toward more innovative tools requires more time. Future studies are necessary to determine if this does occur when the pressure on the care system has lessened.

Although we did not find any differences in general adoption readiness scores, the qualitative data did clearly indicate alterations in practitioners’ perceptions on DMH. Many practitioners explicitly reported to be positively surprised by the possibilities of DMH and to be more convinced of advantages that DMH can offer. Findings of the current study emphasized that providing good care is the primary motivation of practitioners and that they consequently become more open to DMH when they are convinced that this is a means toward that end. This certainly does not imply, however, that practitioners in the future will wish to transfer all their face-to-face contacts to online modes; they generally still prefer face-to-face sessions as their main form of contact and consider adding online components when it has specific added value (e.g., to save travel time or provide homework assignments). This is in line with an earlier study reporting that practitioners prefer hybrid or blended forms of treatment, with 75% of the time face-to-face treatment and 25% online as the ideal distribution (38). Positive results have been reported on blended care, but there exists a wide variation in how it is defined and applied. Ideally, blended treatment involves well-integrated online and offline components that have a well-balanced contribution to the treatment. However, so far, studies show that on- and offline elements are often not truly connected, and online components are used as a supplement to the main face-to-face treatment (39).

For other practitioners, current findings show that the period of mandatory use reinforced a more reluctant attitude toward DMH, and strengthened their preference for having solely face-to-face contact. A main factor in the general preference for face-to-face contact is the experience that remote communication does not allow for satisfying therapeutic interactions and concerns about the negative effects this might have on the therapeutic relationship – a concern that has been consistently reported before as well (2, 3, 16). These concerns were mentioned across the entire sample, although the extent to which practitioners found this problematic to their practice varied substantially between practitioners. Indeed, philosophical criticisms of technology-mediated communication have highlighted its limitations in lack of a shared context and the impossibility of physically sensing the general ambiance in a room and the states and responses of the people present there (40). This author hence concludes that remote communication will always feel like falling short compared to co-present interactions.

Lack of eye contact is also suggested to trigger the experience of impoverished communication (41), as may be the thwarted use of interpersonal distancing, the latter being an important non-verbal behavior to regulate personal space and social interactions (42, 43). As the therapeutic interaction and relationship are crucial factors for successful therapeutic outcomes (6), it seems important to invest future research efforts in deepening our understanding of the exact differences between co-present and remote communication specifically in therapeutic interactions, and to determine the respective benefits and disadvantages of both. Such insights, in turn, could be informative in determining for which therapeutic interventions co-presence is essential, and in which situations remote interaction is sufficient or even advantageous. In addition, this knowledge could guide how current and future DMH tools could be optimized in such a way that they better support therapists in their empathic interactions. Potential directions that are being explored are correcting eye gaze to create direct eye contact or providing therapists with additional information on the client’s arousal level during therapeutic sessions (44).

Another main barrier expressed by multiple respondents involved a strong feeling that DMH is not appropriate for the client group they are treating. Interestingly, opinions on exactly which client group is suitable for DMH were rather mixed. It seems that suitability is very case-specific, as was also suggested by a study of clinicians’ experiences with DMH for autism spectrum disorder (45), and that the complexity in problems experienced by many clients, especially in secondary mental healthcare, does not allow for strict protocolized therapy with predetermined deployment of DMH tools (38). This points to the importance of being flexible in choosing which DMH tools are most appropriate and adapt this to the specific request for help, abilities, and preferences of individual clients, instead of introducing DMH as a one-size-fits-all solution for every client in a particular client group. This also means that not using any DMH tool at all should always be an option as well. Considering the mixed perceptions on the suitability of client groups, more in-depth research is necessary on what works for whom, and at which point in the treatment process. A first review on this topic has recently been published (46). Similarly, suitability of particular tools and correspondent experiences with their use are also likely to be dependent on the specific profession and work setting of the practitioner. For instance, qualitative responses in our study seem to indicate differences between appropriateness of certain DMH tools between in-patient and out-patient settings, but research in in-patient settings is scarce and comparative studies are lacking (47). Further studies on which tools are (most) appropriate in which settings would be valuable to facilitate optimal use of the available DMH interventions.

### 4.2. Considerations for mental healthcare practice

Based on our results, several considerations can be made for the adoption of DMH tools in mental healthcare practice. From our findings, it seems that essential practical requirements such as a sufficient technological infrastructure and procedures concerning privacy and administration have been mostly established during the pandemic, whereas barriers that are related to the treatment itself (i.e., clients’ characteristics and the therapeutic relationship) that are more complex and potentially harder to address have remained and have become more critical now. The knowledge gained to date could be used for the development of best practices or guidelines mapping these characteristics to specific tools to increase therapists’ knowledge on the range of DMH tools and how and when to apply them. Such guidelines should also include contra-indications, providing directions on characteristics that render certain tools less suitable. One instrument has been proposed that might be helpful for making shared decisions on the use of DMH between practitioners and clients (39). Furthermore, just as DMH is not a one-size-fits-all solution for clients, neither is it for practitioners. As has been found in previous work, practitioners perceive particular barriers, drivers, and needs dependent on their position in the adoption process (48). This indicates the importance of designing DMH tools in such a way that they can be customized to one’s individual level of adoption, for example, by offering more basic and advanced versions.

The current study showed that the COVID-19 period seems to have initiated a transition toward more blended forms of treatment, but a sustainable implementation of blended care in mental healthcare practice would require several follow-up steps. An important factor therein is that the rapid technological development of (essential) DMH tools and the increases in practitioners’ skills presented in this study were aimed at enabling the continuation of care under the circumstances of the pandemic. This generally implied transferring the entire treatment to online means. While the gains of this *ad hoc* process are certainly applicable for regular use, sustainable implementation of DMH does come with additional requirements for both the tools as well as the necessary skills. As blended forms with a variable mix of face-to-face and online components are likely to become the default, this also requires practitioners to become proficient in determining when to apply face-to-face or online elements, and how to involve clients in the decision process (38). Implementation of blended care also implies getting accustomed to a different workflow and distribution of time, for example, scheduling time spent on providing feedback on online homework assignments. In addition, now that the extreme pressure of the crisis situation has passed, practitioners should be provided with time to explore how to use the tools in an optimal way, e.g., adjusting the frequency of reminders and feedback. Also crucial for the sustainable implementation of blended care would be to make training on DMH use an integral part of the education for new professionals (e.g., hands-on training with various DMH tools and learning about best practices regarding the integration of these tools in the treatment process).

Regarding DMH tools, further investments are necessary to make them fully integrated in the daily work processes of professionals. An important step therein would be to improve the interoperability of the various available applications to increase ease of use, an important factor in the adoption of innovations (49). Furthermore, improving more innovative tools, such as, Virtual Reality and wearables, and integrating them in DMH platforms can further increase therapists’ options to suit each individual client in an optimal way.

### 4.3. Limitations

Several limitations of the current study should be noted. Although the extraordinary situation of the COVID-19 pandemic offered a unique possibility to learn more about the effect of experience with DMH on the adoption process of professionals, the exceptionality and particular constraints of the circumstances also posed several methodological challenges. First, one of the key rationales for engaging in this study, beyond recording the dramatic changes in DMH tool use itself over the course of a pandemic, was to investigate whether and to what extent first-hand experiences with DMH tools would lead to a lasting change in the use and appreciation of such tools – for example, through increases in self-efficacy or seeing the DMH tools’ effectiveness in interactions with clients. However, the fact that such DMH tool use was largely forced upon the healthcare professional as the only viable option for continued care does raise the question to what extent such experiences generalize to situations of voluntary use. For instance, while during the pandemic the perceived added value of DMH tools was compared to no care – and hence often evaluated as better than nothing - with voluntary use practitioners will take their former (face-to-face) care as their point of comparison, which is likely to alter their estimation of perceived value. Moreover, previous work suggests that a period of involuntary use does not necessarily predict future use (20) and not finding a change in general adoption readiness might be an indication that the altered opinions are only momentary. Future comparative studies are necessary to determine to what extent these changes in use and the intention to continue with blended treatment last when face-to-face contact is entirely unrestricted again.

In addition, this being a cross-sectional study, each survey included different participants, so it cannot be entirely excluded that the found differences are (partly) due to pre-existing differences in characteristics between groups (50). We did, however, apply the same sampling strategy in all three surveys and checked for significant differences in demographic characteristics between the groups. There were none. Furthermore, there were no differences in the distribution of general adoption readiness scores between samples, which further supports the robustness of our findings.

Second, changed frames of reference regarding technology use between studies might also have impacted the results: where formerly meeting in person was the default, remote communication suddenly became the standard during the lockdowns, and this option has become integrated in our way of thinking about social interactions, in our daily lives as well as in mental healthcare practice. Similarly, the widespread demand for digital communication technologies during the pandemic has led to an exceptionally rapid technological development: many applications have become more advanced and new tools have been developed. Hence, what was considered “much use” or “advanced” in the first survey before the pandemic might be considered “average use” and “basic” in the following surveys, potentially biasing the results. More generally, the influence of external factors cannot be fully determined in (repeated) cross-sectional design studies, hence they do not allow to draw strong causal inferences (51).

Last, in the current study, we did not probe which support participants received from their organizations. Even though the specific organizations were mostly similar over the three studies and quantitative results remained the same when controlling for types of organizations and professions, it could be that differences in facilitating conditions between organizations have caused differences in participants’ experiences and perceptions. Similarly, the current study was conducted in the Netherlands and although findings are in line with other recent studies concerning practitioners’ experiences during the COVID-19 pandemic (21), there might be differences with the experiences of practitioners in countries that followed a different policy to regulate the pandemic or where the necessary technological infrastructure to facilitate remote care could not be established. Also, the current study at times probed professionals’ perspectives of clients’ experiences, and so it remains unclear to what extent this aligns with the experiences of clients themselves. Generally, studies show high levels of client satisfaction with DMH, especially when this saves much travel time but, again, this may not be shared by every client as here too substantial interindividual differences in preferences and competences may exist (12). Last, all the data in the current study are based on self-report measures.

## 5. Conclusion

The outbreak of the COVID-19 pandemic in spring 2020 necessitated a sudden, global transition toward the use of DMH tools for mental healthcare delivery. This radical change provided a unique opportunity to analyze how gained experience with DMH tools – albeit out of necessity – influenced practitioners’ adoption of such tools. By having performed similar assessments both before the pandemic and in two periods during the pandemic, the current study yielded a remarkably rich data set that allowed the analysis of the adoption process in a way unparalleled before. Our findings show that the experience gained with DMH during the COVID-19 pandemic has partly changed professionals’ adoption: practitioners have obtained knowledge and skills on how and when to apply several necessary DMH tools and have gained lived experience of a variety of both advantages and disadvantages of DMH tool use in daily practice. The reported intentions on continuing (or discontinuing) blended treatment might indicate a lasting change toward the use of more digitized forms of treatment within mental healthcare. However, considering the sudden and forced use of online therapeutic tools combined with the dramatically increased pressures on the mental healthcare system during the COIVD-19 pandemic, it remains to be seen whether slower, self-initiated changes in the exploration and use of DMH tools under “normal” circumstances will allow for different patterns of DMH adoption to emerge. Either way, further advancements in both DMH technologies as well as professionals’ skills and knowledge regarding a broader set of tools and their integration in the treatment process will be necessary to make blended treatment a success in the long run.

## Data availability statement

The raw data supporting the conclusions of this article will be made available by the authors upon request, without undue reservation.

## Ethics statement

The studies involving human participants were reviewed and approved by the Ethical Review Board of the Human-Technology Interaction Group, Eindhoven University of Technology. The participants provided their written informed consent to participate in this study.

## Author contributions

WI, JB, and MF conceived the study. MF and JB set up and distributed the survey. MF conducted the data analysis and interpretation of the data, and drafted the manuscript. WI, YK, JW, JB, and IB contributed to the critical revision of the manuscript. All authors had seen and approved the submission of this version of the manuscript and took full responsibility for the manuscript.

## Footnotes

1 Even though there were no significant differences in these characteristics, we also performed the analyses with weighted scores for organization type and profession. These analyses yielded no different results.

2 In Survey 3 only, we also added a question on the relative strength with which the barriers, drivers, and needs are experienced, but because the current study focuses on a comparison between the surveys, these results are not reported in this study.

## References

[1] Riper H Andersson G Christensen H Cuijpers P Lange A Eysenbach G. Theme issue on E-mental health: a growing field in internet research. *J Med Internet Res.* (2010) 12:e74. 10.2196/jmir.1713 21169177 PMC3057318

[2] Connolly S Miller C Lindsay J Bauer MS. A systematic review of providers’ attitudes toward telemental health via videoconferencing. *Clin Psychol Sci Pract.* (2020) 27:e12311. 10.1111/cpsp.12311 35966216 PMC9367168

[3] Stoll J Müller J Trachsel M. Ethical issues in online psychotherapy: a narrative review. *Front Psychiatry.* (2020) 10:993. 10.3389/fpsyt.2019.00993 32116819 PMC7026245

[4] Barnett P Goulding L Casetta C Jordan H Sheridan-Rains L Steare T Implementation of telemental health services before COVID-19: rapid umbrella review of systematic reviews. *J Med Internet Res.* (2021) 23:e26492. 10.2196/26492 34061758 PMC8335619

[5] Shigekawa E Fix M Corbett G Roby D Coffman J. The current state of telehealth evidence: a rapid review. *Health Aff.* (2018) 37:1975–82. 10.1377/hlthaff.2018.05132 30633674

[6] Norwood C Moghaddam N Malins S Sabin-Farrell R. Working alliance and outcome effectiveness in videoconferencing psychotherapy: a systematic review and noninferiority meta-analysis. *Clin Psychol Psychother.* (2018) 25:797–808. 10.1002/cpp.2315 30014606

[7] Hennemann S Beutel M Zwerenz R. Ready for eHealth? Health professionals’ acceptance and adoption of eHealth interventions in inpatient routine care. *J Health Commun.* (2017) 22:274–84. 10.1080/10810730.2017.1284286 28248626

[8] Glueckauf R Maheu M Drude K Wells B Wang Y Gustafson D Survey of psychologists’ telebehavioral health practices: technology use, ethical issues, and training needs. *Prof Psychol Res Pract.* (2018) 49:205–19. 10.1037/pro0000188

[9] Van Der Vaart R Atema V Evers A. Guided online self-management interventions in primary care: a survey on use, facilitators, and barriers. *BMC Fam Pract.* (2016) 17:27. 10.1186/s12875-016-0424-0 26961547 PMC4785635

[10] Whitten P Mackert M. Addressing telehealth’s foremost barrier: provider as initial gatekeeper. *Int J Technol Assess Health Care.* (2005) 21:517–21. 10.1017/S0266462305050725 16262977

[11] Ross J Stevenson F Lau R Murray E. Factors that influence the implementation of e-health: a systematic review of systematic reviews (an update). *Implement Sci.* (2016) 11:146. 10.1186/s13012-016-0510-7 27782832 PMC5080780

[12] Vosburg R Robinson K. Telemedicine in primary care during the COVID-19 pandemic: provider and patient satisfaction examined. *Telemed J E Health.* (2022) 28:167–75. 10.1089/tmj.2021.0174 33999740

[13] Nicholas J Bell I Thompson A Valentine L Simsir P Sheppard H Implementation lessons from the transition to telehealth during COVID-19: a survey of clinicians and young people from youth mental health services. *Psychiatry Res.* (2021) 299:113848. 10.1016/j.psychres.2021.113848 33725578 PMC9754759

[14] Rogers E. *Diffusion of innovations.* 5th ed. New York, NY: The Free Press (2003). p. 512.

[15] Feijt M de Kort Y Westerink J Bierbooms J Bongers I IJsselsteijn W. Assessing professionals’ adoption readiness for eMental health: development and validation of the eMental health adoption readiness scale. *J Med Internet Res.* (2021) 23:e28518. 10.2196/28518 34533469 PMC8486999

[16] Davies F Shepherd H Beatty L Clark B Butow P Shaw J. Implementing web-based therapy in routine mental health care: systematic review of health professionals’ perspectives. *J Med Internet Res.* (2020) 22:e17362. 10.2196/17362 32706713 PMC7413287

[17] Vogt E Welch B Bunnell B Barrera J Paige S Owens M Quantifying the impact of COVID-19 on telemedicine utilization: retrospective observational study. *Interact J Med Res.* (2022) 11:e29880. 10.2196/29880 34751158 PMC8797150

[18] Feijt M De Kort Y Bongers I Bierbooms J Westerink J IJsselsteijn W. Mental health care goes online: practitioners’ experiences of providing mental health care during the COVID-19 pandemic. *Cyberpsychol Behav Soc Netw.* (2020) 23:860–4. 10.1089/cyber.2020.0370 32815742

[19] Donovan C Poole C Boyes N Redgate J March S. Australian mental health worker attitudes towards cCBT: what is the role of knowledge? Are there differences? Can we change them?. *Internet Interv.* (2015) 2:372–81. 10.1016/j.invent.2015.09.001

[20] Agarwal R Prasad J. The role of innovation characteristics and perceived voluntariness in the acceptance of information technologies. *Decis Sci.* (1997) 28:557–82. 10.1111/j.1540-5915.1997.tb01322.x

[21] Appleton R Williams J Vera San Juan N Needle J Schlief M Jordan H Implementation, adoption, and perceptions of telemental health during the COVID-19 pandemic: systematic review. *J Med Internet Res.* (2021) 23:e31746. 10.2196/31746 34709179 PMC8664153

[22] De Witte N Carlbring P Etzelmueller A Nordgreen T Karekla M Haddouk L Online consultations in mental healthcare during the COVID-19 outbreak: an international survey study on professionals’ motivations and perceived barriers. *Internet Interv.* (2021) 25:100405. 10.1016/j.invent.2021.100405 34401365 PMC8350604

[23] Coughlin S. Recall bias in epidemiologic studies. *J Clin Epidemiol.* (1990) 43:87–91. 10.1016/0895-4356(90)90060-3 2319285

[24] Jaspers E Lubbers M De Graaf N. Measuring once twice: an evaluation of recalling attitudes in survey research. *Eur Sociol Rev.* (2009) 25:287–301. 10.1093/esr/jcn048

[25] Greene J Caracelli V Graham W. Toward a conceptual framework for mixed-method evaluation designs. *Educ Eval Policy Anal.* (1989) 11:255–74. 10.3102/01623737011003255

[26] Bierbooms J van Haaren M IJsselsteijn W de Kort Y Feijt M Bongers I. Integration of online treatment into the “new normal” in mental health care in post–COVID-19 times: exploratory qualitative study. *JMIR Form Res.* (2020) 4:e21344. 10.2196/21344 33001835 PMC7546865

[27] Delacre M Leys C Mora Y Lakens D. Taking parametric assumptions seriously: arguments for the use of welch’s *F*-test instead of the classical *F*-test in one-way ANOVA. *Int Rev Soc Psychol.* (2019) 32:13. 10.5334/irsp.198

[28] Cohen J. *Statistical power analysis for the behavioral sciences.* 2nd ed. Hillsdale: Lawrence Erlbaum Associates (1988).

[29] Lakens D. Calculating and reporting effect sizes to facilitate cumulative science: a practical primer for t-tests and ANOVAs. *Front Psychol.* (2013) 4:863. 10.3389/fpsyg.2013.00863 24324449 PMC3840331

[30] Hair J Black W Babin B Anderson R. *Multivariate data analysis.* 7th ed. Essex: Pearson (2014).

[31] Fabrigar L Wegener D MacCallum R Strahan E. Evaluating the use of exploratory factor analysis in psychological research. *Psychol Methods.* (1999) 4:272–99. 10.1037/1082-989X.4.3.272

[32] Costello A Osborne J. Best practices in exploratory factor analysis: four recommendations for getting the most from your analysis. *Pract Assess Res Eval.* (2005) 10:1–9. 10.7275/jyj1-4868

[33] Floyd F Widaman K. Factor analysis in the development and refinement of clinical assessment instruments. *Psychol Assess.* (1995) 7:286–99. 10.1037/1040-3590.7.3.286

[34] Finch W. Using fit statistic differences to determine the optimal number of factors to retain in an exploratory factor analysis. *Educ Psychol Meas.* (2020) 80:217–41. 10.1177/0013164419865769 32158020 PMC7047263

[35] Braun V Clarke V. Using thematic analysis in psychology. *Qual Res Psychol.* (2006) 3:77–101. 10.1191/1478088706qp063oa 32100154

[36] Braun V Clarke V Hayfield N Terry G. Thematic analysis. In: Liamputtong P editor. *Handbook of research methods in health social sciences.* Singapore: Springer (2019). p. 843–60. 10.1007/978-981-10-5251-4_103

[37] Muthén L Muthén B. *Mplus user’s guide.* 8th ed. Los Angeles, CA: Muthén & Muthén (1998-2017).

[38] van der Vaart R Witting M Riper H Kooistra L Bohlmeijer E van Gemert-Pijnen L. Blending online therapy into regular face-to-face therapy for depression: content, ratio and preconditions according to patients and therapists using a Delphi study. *BMC Psychiatry.* (2014) 14:355. 10.1186/s12888-014-0355-z 25496393 PMC4271498

[39] Wentzel J van der Vaart R Bohlmeijer E van Gemert-Pijnen J. Mixing online and face-to-face therapy: how to benefit from blended care in mental health care. *JMIR Ment Health.* (2016) 3:e9. 10.2196/mental.4534 26860537 PMC4764785

[40] Dreyfus H. *On the internet.* London: Routledge (2002). 10.4324/9780203754955

[41] Grondin F Lomanowska A Jackson P. Empathy in computer-mediated interactions: a conceptual framework for research and clinical practice. *Clin Psychol Sci Pract.* (2019) 26:17. 10.1111/cpsp.12298

[42] Hayduk L. Personal space: where we now stand. *Psychol Bull.* (1983) 94:293–335. 10.1037/0033-2909.94.2.293

[43] Argyle M Dean J. Eye-contact, distance and affiliation. *Sociometry.* (1965) 28:289. 10.2307/2786027 14341239

[44] Feijt M De Kort Y Westerink J IJsselsteijn W. Enhancing empathic interactions in mental health care : opportunities offered through social interaction technologies. *Annu Rev CyberTherapy Telemed.* (2018) 16:25–31.

[45] Adams L Adamo N Hollocks M Valmaggia L Brewster A Watson J Research in Autism Spectrum Disorders Examining clinicians ’ concerns delivering telemental health interventions directly to autistic individuals during COVID-19. *Res Autism Spectr Disord.* (2022) 94:101956. 10.1016/j.rasd.2022.101956 35369648 PMC8963796

[46] Schlief M Saunders K Appleton R Barnett P Vera San Juan N Foye U Synthesis of the evidence on what works for whom in telemental health: rapid realist review. *Interact J Med Res.* (2022) 11:e38239. 10.2196/38239 35767691 PMC9524537

[47] Sander J Bolinski F Diekmann S Gaebel W Günther K Hauth I Online therapy: an added value for inpatient routine care? Perspectives from mental health care professionals. *Eur Arch Psychiatry Clin Neurosci.* (2022) 272:107–18. 10.1007/s00406-021-01251-1 33725165 PMC7961170

[48] Feijt M de Kort Y Bongers I IJsselsteijn W. Perceived drivers and barriers to the adoption of eMental health by psychologists: the construction of the levels of adoption of eMental health model. . *J Med Internet Res.* (2018) 20:e153. 10.2196/jmir.9485 29691215 PMC5941096

[49] Venkatesh V Thong J Xu X. Consumer acceptance and use of information technology: extending the unified theory of acceptance and use of technology. *MIS Q.* (2012) 36:157. 10.2307/41410412

[50] Rafferty A Walthery P King-Hele S. *Analysing change over time: repeated cross sectional and longitudinal survey data.* Colchester: UK Data Service (2015). p. 25.

[51] Wang X Cheng Z. Cross-sectional studies. *Chest.* (2020) 158:S65–71. 10.1016/j.chest.2020.03.012 32658654

