# Supplementary material for: Integrating technology in mental healthcare practice: A repeated cross-sectional survey study on professionals’ adoption of Digital Mental Health before and during COVID-19
Source: Front Psychiatry. 2023 Feb 16;13:1040023. doi: 10.3389/fpsyt.2022.1040023 (PMC9977803; doi:10.3389/fpsyt.2022.1040023)
Supplement: Supplementary file 1 [file Table_1.docx]

**Supplementary Material 1. Complete Questionnaire.**

## Introduction

## In this questionnaire, the term eHealth is used for all interactions between a mental healthcare provider and client that make use of a technology, e.g., mail, Whatsapp, videoconferencing, eHealth platform, smartphone apps (e.g., for self-monitoring/diary) and Virtual/Augmented Reality.

## Frequency of use, competency, and perceived value regarding eHealth

To which extent do you think that the following eHealth tools are valuable in contact with your clients? (Scale 1 - Not at all to 5 - Very much)

- Telephone (incl. whatsapp call without image)
- Videoconferencing
- E-mail
- Whatsapp / text messaging
- Clientportal
- Online modules in eHealth platform
- Social media (e.g., Facebook)
- Domotics (supporting technology at home)
- Wearables and biofeedback (e.g., heartrate monitor)
- Virtual and/or augmented reality
- 360 degree camera
- Selfmonitoring apps (e.g. sleeping diary, Psymate)
- Online screening (e.g., online intake survey)
- Educational website

How often do you generally make use of each of these eHealth tools in the treatment of your clients? (Scale: 1- (Almost) never, 2 - approximately once every half year, 3 - approximately once every month, 4 - approximately once per week, 5 - (almost) every day)

- Telephone (incl. whatsapp call without image)
- Videoconferencing
- E-mail
- Whatsapp / text messaging
- Clientportal
- Online modules in eHealth platform
- Social media (e.g., Facebook)
- Domotics (supporting technology at home)
- Wearables and biofeedback (e.g., heartrate monitor)
- Virtual and/or augmented reality
- 360 degree camera
- Selfmonitoring apps (e.g. sleeping diary, Psymate)
- Serious gaming
- Online screening (e.g., online intake survey)
- Educational website

Can you indicate how skillful you generally feel when it comes to using eHealth tools? Tick which of the descriptions below suits you best.

- I don't know enough about which eHealth tools are available.
- I have an idea of which eHealth tools are available, but I don't know how to use them.
- I know how to use certain eHealth tools and how to use the basic functionalities.
- I am able to discover for myself how tools work and can therefore work well with various eHealth tools.
- I have good knowledge of various eHealth applications and I am able to transfer my expertise in this area to others.

Below are some statements about your skills in using different tools of eHealth. Indicate for each tool to what extent you think it applies to yourself. (Scale: 1 - Not skilled at all to 5 - Very skilled)

- Telephone (incl. whatsapp call without image)
- Videoconferencing
- E-mail
- Whatsapp / text messaging
- Clientportal
- Online modules in eHealth platform
- Social media (e.g., Facebook)
- Domotics (supporting technology at home)
- Wearables and biofeedback (e.g., heartrate monitor)
- Virtual and/or augmented reality
- 360 degree camera
- Selfmonitoring apps (e.g. sleeping diary, Psymate)
- Serious gaming
- Online screening (e.g., online intake survey)
- Educational website

Below are a some statements about different types of skills that play a role in using eHealth in contact with the client. Indicate per statement to what extent you think it applies to yourself. (Scale: 1 - Strongly disagree to 5 - Strongly agree)

1. I have sufficient knowledge about the possibilities in the field of eHealth to use eHealth tools.
2. I have sufficient ICT and computer skills to use these eHealth applications.
3. I have sufficient knowledge and skills to actively offer eHealth to my clients.
4. When using eHealth I manage to communicate well with my clients.
5. I am aware of the security aspects (privacy and protection of personal data) and considerations in using eHealth in contact with my clients.
6. I am able to deal with the situation that eHealth tools give clients more control when it comes to the content, frequency, form, and location of the contact.
7. I am able to establish an empathetic interaction with my clients in online contact.

## Adoption readiness

Which description do you feel reflects your attitude concerning eHealth in the best way?

- I do not want to start using it
- Using eHealth is not part of my daily routine
- I use what is readily available
- I am exploring more possibilities that are offered by eHealth
- I initiate projects for the further development of eHealth

Indicate per statement to what extent you think it applies to yourself. (Scale: 1- Strongly disagree to 5 - Strongly agree)

1. eHealth fits well to my work as a health care professional
2. Contact between health care professional and client al- ways has to be face-to-face
3. I expect that eHealth provides benefits to the care that I deliver
4. eHealth does not improve the care that I deliver
5. eHealth does not fit the profession of a mental health care professional
6. eHealth does not have any added value for my work as a mental health care professional
7. eHealth is an indispensable part of the mental health care profession
8. I am involved in setting up initiatives for the develop- ment of new eHealth tools and applications
9. Compared to my colleagues, I use eHealth a lot
10. 10. Compared to colleagues, I take a lot of initiative regard- ing eHealth
11. I have ideas about new eHealth tools and technologies that could be developed (eg, Virtual Reality, gaming, biofeedback)
12. In my work I try to stimulate colleagues to use eHealth
13. I have the skills that are necessary to apply eHealth in my work
14. Using eHealth tools comes easy to me
15. I have to learn new skills to start using eHealth

## Barriers, drivers, and needs

Which barriers do you experience when using eHealth? You can tick multiple boxes.

- Problems with the internet connection or overload of systems
- Required software is not available
- Not having the necessary devices (e.g., laptop, camera, headset).
- Clients who do not possess the necessary devices (e.g. laptop, headset, smartphone).
- Clients who do not have the necessary (digital) skills
- Difficulty meeting security and/or privacy requirements (such as GDPR)
- Not knowing how to register treatment hours that are conducted via eHealth tools
- Not being able to perform physical assignments (e.g., use of whiteboard, role-play)
- Difficulty establishing satisfying empathic interactions
- Missing non-verbal cues
- Higher workload
- Resistance of clients
- Difficulty fitting eHealth into the type of treatment I provide
- Difficulties applying eHealth to the client group I treat
- Circumstances in the client's home situation
- Not knowing enough about the possibilities in the field of eHealth
- Not having sufficient digital skills yourself
- Not feeling sufficiently skilled in providing care through EMH
- Insufficient technical support (e.g. ICT helpdesk) from the organization
- Insufficient training and instruction from the organization
- Otherwise, namely
- None of the above

Of these perceived barriers, could you rate how strongly you experience them on a scale from 1 (very mild) to 5 (very strong)?

Which benefits or drivers do you experience when using eHealth? You can tick multiple boxes.

- Reduced travel time for client and/or practitioner
- Increased flexibility in scheduling appointments
- Increased efficiency of appointments, more to-the-point
- Less no-show/higher therapy adherence
- Decreased administration time or shorter team meetings
- Possibility of more frequent short moments of contact
- Client's home environment provides additional information (e.g. tidiness of the house, different behavior in the home situation)
- Increased openness by clients in their own environment
- Client becomes more active
- Client takes more control in his/her treatment
- Lower threshold for care delivery
- Easier to be closely involved with the client
- Better matching with the needs of a (specific) client group
- Possibilities for new forms of treatment
- Otherwise, namely:
- None of the above

Of these perceived benefits, could you rate how strongly you experience them on a scale from 1 (very mild) to 5 (very strong)?

Which needs do you experience regarding the use of eHealth? You can tick multiple boxes.

- Stable internet connection/no delays in video-audio
- Secure (GDPR-adherent) programs
- Better equipment (e.g., laptop, smartphone, headset)
- Additional software features (e.g., online whiteboard, file sharing)
- Support of online group sessions
- Lower pressure on productivity (e.g., fewer clients on a day)
- Easily accessible technical helpdesk
- Guidelines/protocols on procedures for eHealth and recommended software
- Training specifically aimed at eHealth
- Exchange best practices with colleagues
- More user-friendly eHealth tools
- Otherwise, namely:
- None of the above

Of these perceived needs, could you rate how strongly you experience them on a scale from 1 (very mild) to 5 (very strong)?

## Empathic interaction

Below are two statements regarding the empathic interaction with your client. Please indicate per statement to what extent you think it applies to yourself. (Scale: 1- Strongly disagree to 5 - Strongly agree)

1. I manage to establish an empathetic interaction with the client during online contact.
2. Through eHealth I can make contact with the client just as well as face-to-face

Can you indicate why it succeeds or fails to establish a good empathic interaction?

________________________________________________

Which strategies or information do you use to communicate empathically through eHealth?

________________________________________________

## Practitioners' perceptions and experiences on their use of DMH (in response to COVID-19)

How much did you make use of eHealth tools in each of the following periods?

|  | (Almost) never | Approximately once every half year | Approximately once every month | Approximately once per week | (Almost) every day |
| --- | --- | --- | --- | --- | --- |
| Before the COVID-pandemic |  |  |  |  |  |
| During the first lockdown (March-June 2020) |  |  |  |  |  |
| After possibilities for face-to-face contact were expanded again (July 2020 to present) |  |  |  |  |  |

Could you indicate why you are making more or less use of eHealth? ________________________________________________________________

After the COVID-19 restrictions are ended, how much do you expect to continue to use eHealth?

- (Almost) never
- About 1-2 times every six months
- About 1-2 times a month
- About 1-2 times a week
- (Almost) every day

Could you indicate why you expect to continue or stop making use of eHealth? ________________________________________________________________

Could you indicate which specific elements or tools of eHealth you would like to remain using?

________________________________________________________________

How would you rate your current skills regarding eHealth, compared to the respective period:

|  | Much worse | Worse | The same | Better | Much better |
| --- | --- | --- | --- | --- | --- |
| Before the COVID-pandemic |  |  |  |  |  |
| At the end of the first lockdown (March-June 2020) |  |  |  |  |  |

How would you rate the value of eHealth, compared to the respective period:

|  | Much less valuable | Less valuable | Equally valuable | More valuable | Much more valuable |
| --- | --- | --- | --- | --- | --- |
| Before the COVID-pandemic |  |  |  |  |  |
| At the end of the first lockdown (March-June 2020) |  |  |  |  |  |

Could you explain why you find eHealth more or less valuable?

________________________________________________

Could you describe what you think the role of eHealth will be in mental health in the near future?

________________________________________________

## Background questions

What is your gender?

________________________________________________

What is your age?

________________________________________________

What is your highest completed education?

- Primary education
- Secondary education (mavo / havo / vwo)
- Vocational secondary education
- Higher professional education (HBO)
- Scientific education (wo)

What kind of organization do you work in?

- Basic mental healthcare
- Specialist mental healthcare
- General practice
- Independent practice
- Addiction treatment
- General or university hospital
- Forensic institution
- Other, namely: (10) ________________________________________________

What is your profession (in case of multiple positions, choose the one in which you work most of the time)?

- Medical profession (e.g., psychiatrist, doctor)
- Psychotherapeutic profession (psychotherapist)
- Social profession (e.g., social worker, social pedagogical counselor)
- Psychological profession (e.g., primary, child & youth, GZ psychologist)
- Psychological profession - specialism (e.g., clinical (neuro) psychologist)
- Vocational therapeutic profession (e.g., psychomotor therapist, creative therapist)
- Nursing profession (e.g., nurse, social psychiatric nurse)
- Somatic profession (e.g., dietician, physiotherapist, neurologist, general practitioner)
- Supporting profession (e.g., case manager, psychodiagnostics employee, expert by experience)
- Other, namely (23) ________________________________________________

How long have you been working as a healthcare provider? (in years)

________________________________________________

Have you received any education / training to use eHealth tools?

- Yes, during my basic education
- Yes, during a course and / or specialization
- Yes, by following a specific course, webinar or master class for this
- Yes, in another way, namely _______________________________________________
- No
