# Supplementary material for: Integrating technology in mental healthcare practice: A repeated cross-sectional survey study on professionals’ adoption of Digital Mental Health before and during COVID-19
Source: Front Psychiatry. 2023 Feb 16;13:1040023. doi: 10.3389/fpsyt.2022.1040023 (PMC9977803; doi:10.3389/fpsyt.2022.1040023)
Supplement: Supplementary file 2 [file Table_2.docx]

**Supplementary Table 2. Descriptive statistics of general adoption readiness, frequency of use, competency, and perceived value**

|  |  | | Survey 1  M (SD) | Survey 2  M (SD) | Survey 3  M (SD) |
| --- | --- | --- | --- | --- | --- |
| General adoption readiness | | | 3.22 (0.61) | 3.30 (0.60) | 3.29 (0.63) |
| Use | Overall | | 1.73 (0.66) | 2.60 (0.88) | 3.17 (0.84) |
|  |  | E-mail | 3.60 (1.55) | 3.91 (1.3) | 4.00 (1.25) |
|  |  | Text messaging | 2.70 (1.76) | 3.08 (1.77) | 3.32 (1.70) |
|  |  | Videoconferencing | 1.21 (0.76) | 3.48 (1.48) | 3.43 (1.26) |
|  |  | Client portal | 1.77 (1.56) | 2.00 (1.61) | 1.98 (1.52) |
|  |  | Online module | 2.29 (1.39) | 2.30 (1.50) | 2.41 (1.39) |
|  |  | Social media | 1.19 (0.82) | 1.15 (0.71) | 1.21 (0.76) |
|  |  | Monitoring apps | 1.78 (1.24) | 1.92 (1.32) | 1.80 (1.11) |
|  |  | Online screening | 1.12 (1.10) | 1.46 (1.18) | 1.45 (1.06) |
|  |  | Educational website | 2.63 (1.40) | 2.44 (1.24) | 2.57 (1.24) |
|  |  | Domotics | 0.72 (1.10) | 0.79 (0.91) | 1.04 (1.13) |
|  |  | Wearables | 0.92 (1.04) | 0.94 (0.66) | 1.02 (0.66) |
|  |  | VR/AR | 0.84 (0.73) | 0.77 (0.59) | 0.91 (0.64) |
| Competency | Overall | | 2.02 (0.59) | 2.78 (0.80) | 3.18 (0.76) |
|  |  | E-mail | 4.42 (1.03) | 4.75 (0.56) | 4.78 (0.46) |
|  |  | Text messaging | 4.17 (1.31) | 4.47 (1.15) | 4.57 (0.84) |
|  |  | Videoconferencing | 2.25 (1.68) | 2.53 (1.77) | 2.65 (1.63) |
|  |  | Client portal | 3.12 (1.43) | 2.97 (1.59) | 3.11 (1.36) |
|  |  | Online module | 3.00 (1.67) | 3.17 (1.68) | 3.27 (1.65) |
|  |  | Social media | 0.93 (1.30) | 1.27 (1.44) | 1.39 (1.44) |
|  |  | Monitoring apps | 2.88 (1.55) | 4.35 (0.88) | 4.50 (0.65) |
|  |  | Online screening | 1.69 (1.55) | 2.11 (1.57) | 2.15 (1.58) |
|  |  | Educational website | 3.53 (1.49) | 3.62 (1.32) | 3.74 (1.22) |
|  |  | Domotics | 1.30 (1.37) | 1.71 (1.54) | 1.80 (1.48) |
|  |  | Wearables | 1.24 (1.22) | 1.29 (1.35) | 1.38 (1.31) |
|  |  | VR/AR | 2.59 (1.55) | 2.76 (1.56) | 2.79 (1.46) |
| Perceived | Overall | | 2.09 (0.58) | 2.87 (0.73) | 3.37 (0.75) |
| value |  | E-mail | 3.97 (1.07) | 3.95 (1.03) | 3.94 (0.95) |
|  |  | Text messaging | 3.65 (1.31) | 3.51 (1.28) | 3.70 (1.27) |
|  |  | Videoconferencing | 3.25 (1.33) | 4.17 (0.94) | 4.20 (0.90) |
|  |  | Client portal | 3.27 (1.61) | 3.18 (1.58) | 3.34 (1.55) |
|  |  | Online module | 3.97 (1.16) | 3.64 (1.48) | 3.75 (1.33) |
|  |  | Monitoring apps | 3.97 (1.25) | 3.73 (1.36) | 3.91 (1.19) |
|  |  | Online screening | 2.63 (1.75) | 3.00 (1.58) | 3.11 (1.48) |
|  |  | Educational website | 4.04 (1.13) | 3.91 (1.06) | 4.15 (0.95) |
|  |  | Social media | 2.06 (1.12) | 1.79 (1.06) | 2.06 (1.09) |
|  |  | Domotics | 1.77 (1.95) | 2.12 (1.89) | 2.50 (1.83) |
|  |  | Wearables | 2.61 (1.95) | 2.71 (1.64) | 2.94 (1.58) |
|  |  | VR/AR | 2.80 (1.89) | 2.49 (1.85) | 2.89 (1.79) |
