# Supplementary material for: Integrating technology in mental healthcare practice: A repeated cross-sectional survey study on professionals’ adoption of Digital Mental Health before and during COVID-19
Source: Front Psychiatry. 2023 Feb 16;13:1040023. doi: 10.3389/fpsyt.2022.1040023 (PMC9977803; doi:10.3389/fpsyt.2022.1040023)
Supplement: Supplementary file 3 [file Table_3.docx]

**Supplementary Table 3. Post-hoc comparisons of frequency of use, competency, and perceived value of individual DMH tools.**

|  |  | Survey 1-2 | | Survey 1-3 | | Survey 2-3 | |
| --- | --- | --- | --- | --- | --- | --- | --- |
|  |  | Mean diff | Cohen's d | Mean diff | Cohen's d | Mean diff | Cohen's d |
| Use | E-mail | -0.31** | 0.21 | -0.40** | 0.28 | -0.09 | 0.07 |
|  | Text messaging | -0.38** | 0.22 | -0.62** | 0.36 | -0.24 | 0.14 |
|  | Videoconferencing | -2.27** | 1.98 | -2.22** | 2.18 | 0.05 | 0.04 |
|  | Client portal | -0.23 | 0.15 | -0.21 | 0.14 | 0.02 | 0.01 |
|  | Online module | -0.01 | 0.01 | -0.12 | 0.09 | -0.11 | 0.08 |
|  | Social media | 0.04 | 0.05 | -0.02 | 0.03 | -0.06 | 0.08 |
|  | Domotics | -0.14 | 0.11 | -0.02 | 0.02 | 0.12 | 0.10 |
|  | Wearables | -0.34** | 0.30 | -0.33** | 0.31 | 0.01 | 0.01 |
|  | VR/AR | 0.19 | 0.14 | 0.06 | 0.05 | -0.13 | 0.10 |
|  | Monitoring apps | -0.07 | 0.07 | -0.32** | 0.29 | -0.25* | 0.25 |
|  | Online screening | -0.02 | 0.02 | -0.10 | 0.11 | -0.08 | 0.12 |
|  | Educational website | 0.07 | 0.10 | -0.07 | 0.10 | -0.14* | 0.23 |
| Perceived | E-mail | 0.02 | 0.02 | 0.03 | 0.03 | 0.01 | 0.01 |
| value | Text messaging | 0.14 | 0.11 | -0.05 | 0.04 | -0.19 | 0.15 |
|  | Videoconferencing | -0.92** | 0.79 | -0.95** | 0.82 | -0.03 | 0.03 |
|  | Client portal | 0.09 | 0.06 | 0.07 | 0.04 | -0.16 | 0.10 |
|  | Online module | 0.33** | 0.25 | 0.22 | 0.18 | -0.11 | 0.08 |
|  | Social media | 0.27** | 0.25 | 0.00 | 0.00 | -0.27* | 0.25 |
|  | Domotics | 0.24* | 0.18 | 0.06 | 0.05 | -0.18 | 0.14 |
|  | Wearables | -0.37** | 0.22 | -0.48** | 0.29 | -0.11 | 0.07 |
|  | VR/AR | 0.13 | 0.12 | -0.11 | 0.10 | -0.24 | 0.24 |
|  | Monitoring apps | -0.35* | 0.18 | -0.73** | 0.38 | -0.38 | 0.20 |
|  | Online screening | -0.10 | 0.06 | -0.33* | 0.18 | -0.23 | 0.14 |
|  | Educational website | 0.31 | 0.17 | -0.09 | 0.05 | -0.40 | 0.22 |
| Competency | E-mail | -0.33** | 0.39 | -0.36 | 0.44 | -0.03 | 0.06 |
|  | Text messaging | -0.30 | 0.24 | -0.40 | 0.36 | -0.10 | 0.10 |
|  | Videoconferencing | -1.47 | 1.14 | -1.62 | 1.32 | -0.15 | 0.19 |
|  | Client portal | -0.28 | 0.16 | -0.40 | 0.24 | -0.12 | 0.07 |
|  | Online module | 0.15 | 0.10 | 0.01 | 0.01 | -0.14 | 0.09 |
|  | Social media | -0.17 | 0.10 | -0.27 | 0.16 | -0.10 | 0.06 |
|  | Domotics | -0.17 | 0.11 | -0.20 | 0.13 | -0.03 | 0.02 |
|  | Wearables | -0.42 | 0.27 | -0.46 | 0.29 | -0.04 | 0.03 |
|  | VR/AR | -0.09 | 0.06 | -0.21 | 0.15 | -0.12 | 0.09 |
|  | Monitoring apps | -0.34 | 0.25 | -0.46 | 0.34 | -0.12 | 0.08 |
|  | Online screening | -0.41 | 0.28 | -0.50 | 0.35 | -0.09 | 0.06 |
|  | Educational website | -0.05 | 0.04 | -0.14 | 0.11 | -0.09 | 0.07 |
| Note. * = p < 0.05; ** = p < 0.01 | |  |  |  |  |  |  |
