# Supplementary material for: Integrating technology in mental healthcare practice: A repeated cross-sectional survey study on professionals’ adoption of Digital Mental Health before and during COVID-19
Source: Front Psychiatry. 2023 Feb 16;13:1040023. doi: 10.3389/fpsyt.2022.1040023 (PMC9977803; doi:10.3389/fpsyt.2022.1040023)
Supplement: Supplementary file 4 [file Table_4.docx]

**Supplementary Table 4. Factor loadings of final model with 9 factors of barriers, drivers, and needs.**

The first column presents the item, the second column presents the category of the item, and the last 9 columns show the factor loadings for each of the 9 factors. The factor to which the item is assigned in the final model is printed in bold type.

| Item | Category | Connection quality barriers and needs | Equipment availability barriers and needs | Daily work process barriers and needs | Privacy barriers and needs | Empathic interaction barriers | Client-related barriers | Implementation needs | Practical benefits | Client-oriented benefits |
| --- | --- | --- | --- | --- | --- | --- | --- | --- | --- | --- |
| Problems with the internet connection or overload of systems | Barrier | **0.658** | 0.074 | 0.189 | 0.073 | 0.086 | 0.078 | -0.155 | 0.08 | 0.037 |
| Stable internet connection/no lags in image-sound | Need | **0.576** | 0.295 | 0.131 | 0.31 | -0.028 | -0.07 | -0.082 | 0.11 | 0.116 |
| Own lack of necessary devices (e.g., laptop, camera, headset) | Barrier | -0.026 | **0.905** | -0.019 | -0.053 | 0.059 | 0.055 | 0.014 | -0.016 | -0.043 |
| Better equipment (e.g., laptop, smartphone, headset) | Need | 0.166 | **0.629** | -0.017 | 0.086 | -0.108 | 0.021 | 0.118 | 0.027 | 0.02 |
| Required software is not available | Barrier | 0.092 | **0.296** | 0.056 | 0.273 | -0.119 | 0.223 | 0.008 | -0.07 | 0.027 |
| Lack of knowledge on how to register treatment hours conducted via DMH | Barrier | -0.238 | 0.143 | **0.338** | 0.212 | 0.016 | -0.055 | -0.02 | 0.027 | -0.025 |
| Higher workload | Barrier | 0.041 | -0.028 | **0.822** | -0.035 | 0.062 | -0.017 | -0.111 | -0.076 | 0.049 |
| Lower pressure on productivity/less clients on a day | Need | -0.029 | -0.026 | **0.682** | -0.001 | -0.087 | -0.009 | 0.182 | 0.094 | -0.075 |
| Difficulties to adhere to security- and privacy standards (such as GDPR) | Barrier | -0.126 | -0.058 | -0.022 | **0.648** | 0.243 | 0.087 | -0.042 | 0.097 | -0.049 |
| Secured (GDPR-proof) software | Need | 0.113 | 0 | -0.006 | **0.801** | -0.03 | -0.036 | 0.087 | -0.023 | -0.038 |
| Difficulties to establish a satisfactory empathic interaction | Barrier | -0.058 | 0.077 | -0.009 | -0.021 | **0.92** | -0.026 | 0.012 | -0.003 | 0.038 |
| Missing non-verbal cues | Barrier | 0.119 | -0.093 | 0.038 | 0.113 | **0.762** | -0.029 | 0.05 | 0.021 | -0.036 |
| Clients who do not possess the necessary devices (e.g., laptop, headset, smartphone) | Barrier | 0.11 | 0.128 | -0.071 | -0.092 | -0.048 | **0.753** | -0.011 | 0.003 | 0.019 |
| Clients who do not have the necessary (digital) skills | Barrier | -0.1 | 0.011 | 0.007 | 0.08 | -0.052 | **0.837** | -0.02 | -0.014 | 0.014 |
| Resistance of clients | Barrier | 0.024 | -0.158 | 0.163 | -0.043 | 0.189 | **0.455** | 0.147 | -0.06 | -0.068 |
| Circumstances in the home environment of clients | Barrier | 0.443 | -0.216 | -0.042 | -0.036 | 0.173 | **0.33** | 0.115 | 0.038 | -0.042 |
| Additional possibilities of software (e.g., online whiteboard, sharing files) | Need | 0.302 | -0.03 | 0.069 | 0.137 | -0.15 | -0.013 | **0.385** | 0.15 | -0.148 |
| Facilitation of group sessions | Need | 0.325 | 0.032 | 0.005 | -0.094 | -0.026 | 0.005 | **0.392** | 0.106 | 0.131 |
| Guidelines/protocols on procedures in online treatments and recommended software | Need | 0.006 | -0.055 | -0.043 | 0.3 | 0.017 | -0.062 | **0.529** | -0.038 | 0.034 |
| Training specifically focused on online treatment | Need | -0.118 | 0.121 | -0.051 | 0.014 | 0.081 | 0.015 | **0.716** | -0.037 | 0.05 |
| Exchange of best practices with peers | Need | -0.094 | -0.007 | 0.136 | -0.001 | 0.091 | 0.035 | **0.55** | 0.101 | 0.079 |
| Easily available technical helpdesk | Need | -0.083 | 0.136 | 0.243 | 0.331 | 0.012 | 0.156 | **0.218** | 0.143 | 0.132 |
| Reduced travel time for client and/or therapist | Driver | 0.246 | 0.014 | 0.114 | -0.03 | 0.121 | 0.075 | 0.013 | **0.722** | -0.15 |
| More flexibility in scheduling appointments | Driver | -0.003 | -0.08 | -0.03 | 0.058 | -0.025 | -0.075 | -0.005 | **0.643** | 0.015 |
| More efficient sessions, more to-the-point | Driver | -0.163 | 0.086 | -0.023 | 0.031 | -0.081 | -0.029 | 0.002 | **0.622** | 0.137 |
| Less no-show/higher treatment adherence | Driver | -0.074 | 0.015 | -0.02 | -0.088 | -0.029 | -0.157 | 0.14 | **0.608** | 0.045 |
| Faster administration or staff meetings | Driver | -0.03 | -0.046 | -0.197 | 0.132 | -0.019 | 0.09 | -0.211 | **0.552** | 0.056 |
| Possibility of more frequent short moments of contact in-between sessions | Driver | -0.176 | -0.098 | 0.022 | 0.045 | -0.015 | 0.118 | 0.095 | **0.394** | 0.099 |
| Home environment of client provides supplementary information (e.g., tidiness of room, other behavior in home environment) | Driver | 0.336 | -0.24 | -0.051 | 0.104 | -0.077 | -0.035 | 0.198 | 0.119 | **0.301** |
| Increased openness of clients in their own environment | Driver | 0.023 | -0.016 | 0.005 | -0.024 | 0.02 | 0.008 | 0.011 | -0.008 | **1.12** |
| Client becomes more active, take more ownership | Driver | -0.329 | 0.017 | -0.017 | 0.048 | -0.247 | 0.012 | 0.006 | 0.061 | **0.358** |
